# Insufficient Knowledge of Breast Cancer Risk Factors Among Malaysian Female University Students

**DOI:** 10.5539/gjhs.v8n1p277

**Published:** 2015-07-30

**Authors:** Asnarulkhadi Abu Samah, Maryam Ahmadian, Latiffah A. Latiff

**Affiliations:** 1 Institute for Social Science Studies, Universiti Putra Malaysia (UPM), 43400, UPM Serdang, Selangor, Malaysia; 2 Cancer Resource and Education Centre, Faculty of Medicine and Health Sciences, 43400, UPM Serdang, Selangor, Malaysia

**Keywords:** breast cancer, breast self-examination, knowledge, signs and symptoms, risk factors, Malaysia

## Abstract

**Background:**

Despite continuous argument about the efficacy of breast self-examination; it still could be a life-saving technique through inspiring and empowering women to take better control over their body/breast and health. This study investigated Malaysian female university students’ knowledge about breast cancer risk factors, signs, and symptoms and assessed breast self-examination frequency among students.

**Method:**

A cross-sectional survey was conducted in 2013 in nine public and private universities in the Klang Valley and Selangor. 842 female students were respondents for the self-administered survey technique. Simple descriptive and inferential statistics were employed for data analysis.

**Results:**

The uptake of breast self-examination (BSE) was less than 50% among the students. Most of students had insufficient knowledge on several breast cancer risk factors.

**Conclusion:**

Actions and efforts should be done to increase knowledge of breast cancer through the development of ethnically and traditionally sensitive educational training on BSE and breast cancer literacy.

## 1. Introduction

Developing countries are observing alarming rates of breast cancer, particularly among young women (Ahmadian and Abu Samah, 2014). In Malaysia, breast cancer is the most common cancer among women. Five thousands four hundred and ten cases were reported in 2014 which accounted for 24.5% of total cancer related deaths in Malaysia for 2014 (World Health Organization, 2014). Personal motivation and individual’s engagement in preventive actions among Asian women is a timely action (Ahmadian & Abu Samah, 2013).

Not only the advantages associated with early detection of breast cancer and cognitive factors influencing preventive behaviors for breast cancer but also women’s knowledge on breast cancer signs, symptoms and risk factors is critical to the improvement of breast cancer interventions. Despite a few studies on breast cancer literacy in Malaysia, our knowledge about breast cancer knowledge of female university students in Malaysia is imperceptible.

A recent study carried out among 10,242 undergraduate university students in 24 countries across Asia, Africa and America revealed that 35.4% of the women were not aware of any risk factors (e.g. heredity, alcohol, exercise, overweight, stress, smoking, dietary fat and fibre) influencing breast cancer (Peltzer & Pengpid, 2014).

In Malaysia, educational level influences the knowledge of breast cancer signs, symptoms, and risk factors. However, breast cancer literacy is not the same in several ethnicities among Malaysian women. For instance, a study on 320 women from different age groups and various ethnicities in Sungai Petani, Kedah revealed that Chinese women had a better knowledge towards breast cancer, but Malay women had a better knowledge on the risk factors of breast cancer (Baig et al., 2011).

Ethnicity, employment status, and educational level were also the strongest predictors of breast cancer knowledge and literacy (Hadi et al., 2010). Previous study among 544 respondents in Kedah and Perlis, Malaysia presented that the majority of participants with tertiary education have knowledge on cancer risk factors (Samat et al., 2014). In contrast, a cross sectional survey done among 500 females aged 15–19 years old in Malaysia demonstrated an inadequate knowledge level of breast self-examination and risk factors for breast cancer (Che et al., 2014).

Further, breast cancer is a progressive disease, small tumours are more likely to be at an early stage, and therefore early detection is more likely to have more effective treatment and a better prognosis (Tabar et al., 1999). Literature showed that breast self-examination is also fascinating as a patient-centred and non-invasive screening procedure that allows women to examine their own body in a relaxed manner (Vainio & Bianchini, 2002). Thus, information on early detection, particularly breast self–examination is very important to reduce the mortality rates among young women (Smith et al., 2003; Tabar et al., 1999).

Given the previous literature on the frequency of breast self-examination and knowledge of breast cancer, signs, symptoms and risk factors; breast cancer literacy might be a noticeable factor in cancer early detection. Though, information is still limited regarding differences in women’s breast cancer literacy and self-exam practices by several socio-demographic status, location, and specific racial/ethnic groups in Malaysia. This study aimed to evaluate breast cancer literacy among young female university students in the Klang Valley and Selangor, Malaysia. The main goal of the study is the need for the development of a new assessment tool for breast cancer literacy and to present tailored intervention plans and programs in the future. The most important ambition of the study is to involve health care providers in decision making process for the development of breast cancer education for low-literacy women in Southeast Asia.

## 2. Methodology

### 2.1 Setting

The participants in the study were female students in the selected public and private universities in the Klang Valley and Selangor, Malaysia. Details of this study has already been published somewhere else (Abu Samah & Ahmadian, 2014). In summary, a multistage cluster random sampling technique was used to select the participants in the selected universities between the months of November and December, 2013. 842 (99%) Out of 850 questionnaires were successfully retrieved. The study was sponsored by the University Research Grant Scheme and approved by the University Research Ethics’ Committee. Informed consent was obtained from all students.

The initial sample size was assessed based on the number of students at the designated universities with absolute precision of 5%. The participants aged ranges between 17 - 52 years old (M=22.51; SD= 4.82). Majority of the participants were undergraduate students (84.6%).

### 2.2 Data Collection

A self-administered paper questionnaire was applied to collect the data from students after explaining the survey purpose. The questionnaire was adopted and adapted from other published studies dealing with same subject matter (Abu Samah & Ahmadian, 2014; Alharbi et al., 2012; Chait et al., 2009; Chouliara et al., 2004; Luszczynska & Schwarzer, 2003; Sung et al., 1997). Questionnaires were made anonymous due to privacy-related issues.

Socio-demographic characteristic used in this study were age, education level, major of study, marital status, occupation, monthly family income, race, religion, and family history of breast cancer and etc. The frequency of breast self-examination (BSE) was asked for two different situations: BSE in the past year and intention to practice BSE in the next year. Knowledge about the risk of breast cancer along with breast cancer signs and symptoms were assessed by selected questionnaire items. A positive answer was assigned one point, whereas a negative answer was given zero.

### 2.3 Data Analysis

The Statistical Package for Social Sciences (SPSS21) was used for data processing. Simple descriptive and inferential statistics were used for data analysis and frequency with percentage distribution applied for categorized variables. Using the median value as a cut-off point, respondents were categorized into two groups: low level group (with scores less than the median) and satisfactory level group (with scores equal or higher than the median) (Alharbi et al., 2012). The median value of the percentage score of all the participants was 52.6 for breast cancer risk factors and 69.45 for signs and symptoms.

## 3. Results & Discussion

### 3.1 Distribution of Respondents

The study participants consisted of 842 Malaysian female university students which is majority of them were aged below 29 years old (92.3%) and were undergraduate students (84.6%) (Table 1). Most of the participants were studying in social sciences and art (55%), 38.6% were studying life sciences, physical sciences and engineering and the rest of them were in medical and health care sciences field. In terms of marital status, the majority of the participants were single (89.7%) and only 9.1% of the participants were married or ever married.

**Table 1:** Socio-demographic characteristics of respondents (n=842)

Variables	No	%
Age groups		
≤ 29	777	92.3
30-39	47	5.60
≥ 40	18	2.10
Highest Level of Education		
Diploma	0	0
Undergraduate Students	712	84.6
Master’s Students	109	12.9
Ph.D. Students	21	2.50
Major of study		
Life sciences, Physical sciences, and Engineering	307	38.6
Social sciences and Art	438	55
Medical and Health care sciences	51	6.40
Marital status		
Married	70	8.30
widow	2	0.20
divorced	5	0.60
Single	755	89.7
Others	10	1.20
Length of marriage		
1-5	33	50
6-10	14	21.2
11-15	10	15.2
16-20	6	9.10
≥21	3	4.50
Occupational status		
Full-time Student (no occupation)	731	86.8
Part Time Employee	24	2.90
Full time employee	56	6.70
Housewife	4	0.50
Others	27	3.20
Place of origin		
Urban areas	540	64.1
Rural areas	302	35.9
Monthly family income		
<RM2500	467	55.5
RM2500 – RM7500	302	35.9
>RM7500	73	8.70
Race		
Malay	554	65.8
Chinese	182	21.6
Indian	72	8.60
Other	34	4.00
Religion		
Islam	569	67.6
Buddha	133	15.8
Christian	53	6.30
Hindu	66	7.80
Other	21	2.50
Health insurance status		
Public	185	23.2
Private	317	39.7
Uninsured	297	37.2
Smoking status		
Smoker	3	0.40
Ex-smoker	5	0.60
Non-smoker	829	99.0
Normal menstrual cycle		
Yes	675	80.5
No	164	19.5
Abortion history		
Yes	19	2.30
No	821	97.7
Breast feeding history		
Yes	63	7.50
No	777	92.5
Method of contraceptive use		
None	780	94.7
Pills	12	1.50
Others	32	3.90
Family History of Breast Cancer		
Yes	100	11.9
No	742	88.1

Most of the participants (86.8%) have no occupation which means they were full-time students while 9.6% of the participants were working. 64.1% of the participants were from urban areas and more than half of all the participants had monthly family income less than RM2500 (55.5%). In terms of ethnic background, Malay comprised the majority of the participants (65.8%), followed by Chinese (21.6%), and then Indian (8.60%). 62.9% of the participants admitted that they have a health insurance.

The majority of the participants (99%) stated that they were not smoking at all. Among the participants, 675 (80.5%) had a normal menstrual cycle, 19 (2.3%) had an abortion history, and 63 (7.5%) had a breastfeeding history. The percentage of students with a family history of breast cancer (11.9%) in the study is also considerable (Table 1).

### 3.2 Breast Self-exam Behaviors and Intentions

Breast Self-Examination (BSE) was asked for two different periods; breast self-examination practice (BSE) in the last year and intention to conduct breast self-examination (BSE) in the next year. Out of 842 participants, 54.5 % (459) “never applied” BSE in the past year and 32.5% (274) applied BSE “whenever it came to their mind”. In addition, 18.5% (156) of the respondents declared no intention to practice BSE in the next year and 25.2% (212) of them admitted that they will apply BSE monthly.

With regard to BSE in the past, the ANOVA results showed that there was statistically significant differences between participants regarding different levels of education, ethnicity, and age groups (P<0.05). The ANOVA analysis also revealed that there was a significant difference in race between Malay, Chinese and Indian and age groups on BSE intensions (P<0.05). The results of the ANOVA tables for demographic characteristics and BSE behaviors and intentions have already been published in our recent study (Abu Samah and Ahmadian, 2014). In fact, the ANOVA results exposed that education, race and age group could influence past breast self-exams behaviors and intentions to conduct barest self-exams in the future. The ANOVA results uncovered that Indian students had the higher mean score among the groups with regard to past breast self-exams while Malaysian students had a greater mean score for intention to conduct breast self-exams in the future. The study discovered that Masters’ students had the highest mean score for past breast self-exams behaviors. The findings also confirmed that the students aged between 30 to 40 years old not only had the higher mean score for past breast self-exams but also reported a higher mean score for intention to do breast self-exams in the future. However, the results might be an overstatement of the value of the analysis (Ahmadian et al., 2012).

The rate of BSE (45.5%) is the same as the percentage of BSE in the other studies such as Turkish female teachers (43.9%), and Turkish midwives (52%) (Nur, 2010; Ertem & Koçer, 2009). However, this rate is much higher than the percentage of BSE (21%) in previous studies conducted on Kuwaiti female teachers (Alharbi et al., 2012) and women who attended primary health clinics in Kuwait (Al-Azmy et al., 2013).

In contrast to our results, another study reported that Malay women were more likely to perform BSE (Dunn & Tan, 2011). Besides, the uptake of BSE among Malaysian women in 2006 was more than 50% (Institute of Health, 2008; Dahlui et al., 2013). In view of the results on BSE behaviors, implications for education/training of female students on breast health and BSE practice are recommended. However, we believe that actual community participation in heath does not simply imply taking part in an action planned by health care professionals in a top-down approach and students/women should also decide on their health prioritization such as breast screening behaviors (Ahmadian & Abu Samah, 2012).

### 3.3 Risk Factors, Signs and Symptoms of Breast Cancer

Participant’s knowledge about items related to risk factors of breast cancer was asked. The percentages of those responded correctly for each items were presented in Table 2. Study showed that 88.8% of all the participants answered correctly about the family history of cancer/heredity. 76.5% answered correctly about radiation exposure, 68.3% about cigarette smoking and occupational risk factors. 64.4% of the participants answered correctly about eating fruits and vegetables/fibre. The percentages were 63.9% for consumption of fatty foods and 60.6% for a stressful life. However, only 29% of the participants answered correctly about breast feeding and 20.8% of the participants answered correctly about early children.

**Table 2:** Percentage of respondents with correct knowledge about items related to risk factors, signs and symptoms of breast cancer (n=842)

Risk Factors	No	%
Cigarette smoking	575	68.3
Alcohol drink	493	58.6
Hormone replacement therapy	483	57.4
A stressful life	510	60.6
Regular exercise/physical activity	464	55.1
Eating fruits and vegetables/fiber	542	64.4
Early children	175	20.8
Aging	422	50.1
Consumption of fatty foods	538	63.9
Consumption of spicy foods	237	28.1
Radiation exposure	644	76.5
Having benign breast disease	35	4.20
Family history of cancer/heredity	744	88.8
Personal hygiene	164	19.5
Oral contraceptives	264	31.4
Early onset of menses (before the age of 12 years)	207	24.6
Late menopause (after the age of 55 years)	218	25.9
Breast feeding	244	29.0
Overweight	346	41.1
Occupational risk factors	575	68.3
*Sign and symptoms*		
Bloody discharge from nipple	593	70.4
Breast mass	514	61.0
Breast pain	97	11.5
Enlargement of neighboring lymph nodes	666	79.1
Lump in armpit	662	78.6
Limp in breast	752	89.3
Breast skin retraction	557	66.2
Abnormal arm swelling	503	59.7
Nipple retraction	627	74.5
Discoloration of breast	577	68.5

Meanwhile, 28.1% of the participant answered correctly about consumption of spicy foods. A small number of participants replied correctly on personal hygiene (19.5%) and having benign breast disease (4.2%).

Participants showed deprived understanding of major breast cancer risk factors such as over –weight, oral contraceptive, breast feeding, late menopause, and early onset of menses. Similarly, in another study among Muslim women in the Middle East, participants had also limited knowledge about breast cancer risk factors such as obesity, oral contraceptive, late menopause, and early onset of menses (Alharbi et al., 2012). Previous studies also cited women’s limited knowledge about breast cancer risk factors (Che et al., 2014; Alharbi et al., 2012; Leslie et al., 2003; Abdel-Fattah et al., 2000; Breslow et al., 1997).

Our results on students’ knowledge about the risk factors of breast cancer also supported the interpretation of why 54.5 % (459) of female university students in the study never applied BSE in the last year.

The percentages of those replied correctly for each items of breast cancer signs and symptoms were also presented in Table 2. The percentages for this section was more than 69.45(cut-off) in which most participants (89.3%) answered correctly about lump in breast, 79.1% for enlargement of neighbouring lymph nodes, 78.6% for lump in armpit, nipple retraction (74.5%) and 70.4% for bloody discharge from nipple. However, only 11.5% answered correctly about breast pain. The knowledge about breast pain as a sign of breast cancer was found to be poor among the participants and approximately 88% of them were supposed that breast pain is one of the symptoms for breast cancer.

## 5. Conclusion

The uptake of breast self-examination (BSE) was less than 50% among selected students. Results also portrayed that 32.5% of the respondents did not routinely perform breast self-exams and most of the students had insufficient knowledge on several breast cancer risk factors. However, students’ knowledge on breast cancer signs and symptoms were higher than median score. The results of this paper denote that it is not an easy task to focus on breast cancer prevention/screening, even among educated people in Malaysia the same as a number of other countries in the Southeast Asia region.

The findings on breast cancer literacy highlighted the need for a new assessment tool for Muslim women in Southeast Asia to advocate their adherence to breast cancer screening standards. Besides, new approaches to cancer prevention programs should involve the targeted audiences (e.g. the uneducated, half educated and so-called educated women) in pursuing to change their wrong perceptions, negative cultural beliefs and social taboos about cancer.

This analysis will postulate health care providers with the conceptual basis to realistically compare breast self-examination frequency and cancer literacy among various populations while also serving as a guide for further tailored interventions for breast cancer prevention among young women. A larger sample from rural and urban areas in others states in Malaysia should be included in the others studies to investigate the differences and similarities among varied ethnicities and age groups of women to develop an exclusive breast cancer literacy assessment tool.

We do accept that this study characterised with some limitations. On the contrary of this study, the majority of breast cancer studies regarding breast self-exams believed these exams as a part of treatment for patients suffering from breast diseases. Similar to other studies, we used to implement the adaptive questionnaire design in the survey. Our respondents were more under-graduates, quite fluent in English, and from urban areas. Nevertheless, the implication of the study would be the need for appropriate interventions/campaigns to raise the women’s awareness of breast cancer signs, symptoms, risk factors, and common misconceptions about the respective items.

## References

[ref1] Abdel-Fattah M, Zaki A, Bassili A, Shazly M, Tognoni G (2000). Breast self-examination practice and its impact on breast cancer diagnosis in Alexandria, Egypt. East Mediterr Health J.

[ref2] Abu Samah A, Ahmadian M (2014). Relationship between Body Image and Breast Self-examination Intentions and Behavior among Female University Students in Malaysia. Asian Pac J Cancer Prev.

[ref3] Ahmadian M, Abu Samah A (2012). A model for community participation in breast cancer prevention in Iran. Asian Pac J Cancer Prev.

[ref4] Ahmadian M, Abu Samah A (2013). Application of healthbehavior theories to breast cancer screening among Asian women. Asian Pac J Cancer Prev.

[ref5] Ahmadian M, Abu Samah A (2014). An outline of the need for psychology knowledge in health professionals: implications for community development and breast cancer prevention. Asian Pac J Cancer Prev.

[ref6] Ahmadian M, Abu Samah A, Redzuan M. R, Emby Z (2012). Predictors of mammography screening among Iranian women attending outpatient clinics in Tehran, Iran. Asian Pac J Cancer Prev.

[ref7] Al-Azmy S. F, Alkhabbaz A, Almutawa H. A (2013). Practicing breast self-examination among women attending primary health care in Kuwait. Alexandria J of Med.

[ref8] Alharbi N. A, Alshammari M. S, Almutairi B. M, Makboul G, El-Shazly M. K (2012). Knowledge, awareness, and practices concerning breast cancer among Kuwaiti female school teachers. Alexandria J of Med.

[ref9] Baig M. R, Subramaniam V, Chandrasegar A. A, Khan T. M (2011). A population based survey on knowledge and awareness of breast cancer in the suburban females of Sungai Petani, Kedah, Malaysia. Int J Oncol Res on Internal Med & Pub Health.

[ref10] Breslow R. A, Sorkin J. D, Frey C. M, Kessler L. G (1997). Americans' knowledge of cancer risk and survival. Prev Med.

[ref11] Chait S. R, Thompson J. K, Jacobsen P. B (2009). Relationship of body image to breast and skin self-examination intentions and behaviors. Body image.

[ref12] Che C. C, Coomarasamy J. D, Suppayah B (2014). Perception of Breast Health amongst Malaysian Female Adolescents. Asian Pac J Cancer Prev.

[ref13] Chouliara Z, Papadioti-Athanasiou V, Power K, Swanson V (2004). Practice of and attitudes toward breast self-examination (BSE): A cross-cultural comparison between younger women in Scotland and Greece. Health Care Women Int.

[ref14] Dahlui M, Gan D. E. H, Taib N. A, Lim J. N. W (2013). Breast screening and health issues among rural females in Malaysia: How much do they know and practice?. Prev Med.

[ref15] Dunn R. A, Tan A. K (2011). Utilization of breast cancer screening methods in a developing nation: Results from a nationally representative sample of Malaysian households. Breast J.

[ref16] Ertem G, Koçer A (2009). Breast self-examination among nurses and midwives in Odemis health district in Turkey. Indian J Cancer.

[ref17] Hadi M. A, Hassali M. A, Shafie A. A, Awaisu A (2010). Knowledge and perception of breast cancer among women of various ethnic groups in the state of Penang: A cross-sectional survey. Med Princ Pract.

[ref18] Institute Health f.P (2008). National Health and Morbidity Survey 2006 Breast cancer.

[ref19] Leslie N. S, Deiriggi P, Gross S, DuRant M. E, Smith C, Veshnesky J. G (2003). Knowledge, attitudes, and practices surrounding breast cancer screening in educated Appalachian women. Oncol Nurs Forum.

[ref20] Luszczynska A, Schwarzer R (2003). Planning and self-efficacy in the adoption and maintenance of breast self-examination: A longitudinal study on self-regulatory cognitions. Psychol Health.

[ref21] Nur N (2010). Breast cancer knowledge and screening behaviors of the female teachers. Women Health.

[ref22] Peltzer K, Pengpid S (2014). Awareness of Breast Cancer Risk among Female University Students from 24 Low, Middle Income and Emerging Economy Countries. Asian Pac J Cancer Prev.

[ref23] Samat N, Ghazali S, Atang C (2014). Awareness and Knowledge of Cancer: A Community Survey in Kedah and Perlis. ASS.

[ref24] Smith R. A, Saslow D, Sawyer K. A (2003). American Cancer Society guidelines for breast cancer screening. CA Cancer J Clin.

[ref25] Sung J. F, Blumenthal D. S, Coates R. J, Alema-Mensah E (1997). Knowledge, beliefs, attitudes, and cancer screening among inner-city African-American women. Journal of the National Medical Association.

[ref26] Tabar L, Duffy S. W, Vitak B, Chen H. H, Prevost T. C (1999). The natural history of breast carcinoma: what have we learned from screening?. Cancer.

[ref27] Vainio H, Bianchini F (2002). Breast cancer screening: International Agency for Research on Cancer (IARC) handbooks of cancer prevention.

